# Forecast accuracy of demand for registered nurses and its determinants in South Korea

**DOI:** 10.1186/s12960-024-00910-3

**Published:** 2024-06-25

**Authors:** Suyong Jeong

**Affiliations:** Department of Nursing, College of Health and Welfare, Gangneung-Wonju National University, 26403 Wonju-Si, Gangwon-Do, Republic of Korea

**Keywords:** Nursing workforce, Demand forecasting, Forecast accuracy, Human resources

## Abstract

**Background:**

Despite the significance of demand forecasting accuracy for the registered nurse (RN) workforce, few studies have evaluated past forecasts.

**Purpose:**

This paper examined the ex post accuracy of past forecasting studies focusing on RN demand and explored its determinants on the accuracy of demand forecasts.

**Methods:**

Data were collected by systematically reviewing national reports or articles on RN demand forecasts. The mean absolute percentage error (MAPE) was measured for forecasting error by comparing the forecast with the actual demand (employed RNs). Nonparametric tests, the Mann‒Whitney test, and the Kruskal‒Wallis test were used to analyze the differences in the MAPE according to the variables, which are methodological and researcher factors.

**Results:**

A total of 105 forecast horizons and 196 forecasts were analyzed. The average MAPE of the total forecast horizon was 34.8%. Among the methodological factors, the most common determinant affecting forecast accuracy was the RN productivity assumption. The longer the length of the forecast horizon was, the greater the MAPE was. The longer the length of the data period was, the greater the MAPE was. Moreover, there was no significant difference among the researchers’ factors.

**Conclusions:**

To improve demand forecast accuracy, future studies need to accurately measure RN workload and productivity in a manner consistent with the real world.

**Supplementary Information:**

The online version contains supplementary material available at 10.1186/s12960-024-00910-3.

## Introduction

Nurses are among the most significant contributors to human health, and a large body of related research has focused on their impact on patient safety and their increasing roles and responsibilities in modern society [1–4]. Human resource planning for nursing personnel has emerged as an important policy issue in terms of preventing disease, improving health, and reducing medical costs. A forecast is a description of the future state that a forecaster creates using a conscious reasoning system [5]. This is a very challenging task not only because many variables affect the future state, but also because the interrelationships among these variables are extensive. Nevertheless, forecasting is important for detecting sources of future uncertainty early and minimizing future risk. When applied to human health resources, forecasting practices guide health workforce planning and impact the quality of health care delivery.

In 2021, South Korea’s health care spending surged to approximately 142 billion U.S. dollars, marking a remarkable 5.4-fold increase since 2003, indicating rapid growth [6]. Despite the predominant reliance on public financing, which accounts for 62.3% of total spending through sources such as social insurance and government funds, the private sector contributes 37.7% mainly through out-of-pocket expenses and private insurance [6]. Notably, private hospitals dominate service provision, constituting approximately 90% of health care services, whereas public hospitals represent only 10% [7]. Universal health insurance in South Korea significantly reduces costs and enhances health care utilization rates. Consequently, health care providers tend to prioritize acute care services in private hospitals over public health initiatives. This disparity is also evident in the distribution of nursing personnel across health care institutions. As of 2020, South Korea had a total of 391,493 nurses, with 55.3% employed in clinical sectors, 17.5% in nonclinical sectors, and 27.2% in inactive sectors [8]. Hospital nurses constitute 90.4% of the workforce in clinical sectors, whereas nurses in ambulatory clinics and public health center nurses account for 6.4% and 1.5%, respectively [8]. Nurses employed in long-term care facilities, categorized as nonclinical sectors in South Korea, comprise only 6% of total nonclinical workers [8].

Forecasting workforce demand involves estimating the quantity and types of human resources an organization will require in the future. For registered nurses (RNs), demand forecasting aims to predict the number and types of nurses needed in the future. Although demand, requirements and needs are distinct concepts, forecasting researchers tend to use them synonymously. However, from an economic standpoint, RN demand represents the labor that health care institutions are willing to purchase in the market at a wage level, which is derived from society's health care demands [9]. Conversely, requirements or needs, irrespective of economic considerations, denote the labor necessary for a society to achieve a certain health benchmark, which is determined subjectively [9]. RN demand forecasting pertains to the labor market where nurses are employed, whereas RN supply forecasting is linked to educational and training institutions that produce nurses. Supply forecasting for RNs is comparatively straightforward and accurate given its fewer variables and less variability. In contrast, demand forecasting for RNs is more complex and less accurate due to the multitude of variables and their high interdependence, requiring a sophisticated forecasting process.

In their study, Hall and Mejía proposed four typical approaches for forecasting the health care workforce [10]. The first approach, known as the “health needs” method, aims to meet the optimal level of service corresponding to the nation’s health needs, regardless of medical costs or available service supply. It estimates personnel based on normative judgments of service requirements and can be seen as encompassing the “potential demand” for health care services. The second approach, termed the “service target” method, sets a target service level based on specific service production and delivery standards. It estimates that the workforce required to achieve these targets depends on health-related policies and goals and shares normative characteristics similar to those of the health needs method. The third approach, referred to as the “health demands” method, predicts workforce levels based on “economic effective demand”. According to Parnes [11], this approach utilizes quantitative economic techniques to gradually estimate workforce demand by modeling to achieve the targeted total production at the national level, using the labor–output ratio as a crucial variable. The fourth approach, known as the “manpower population ratio” method, assesses workforce adequacy based on the ratio of desired health care personnel to the population. Although this method is often used as a complementary approach in workforce demand prediction studies due to its simplicity, it fails to explain productivity changes or dynamic workforce supply factors. The primary methodology used in forecasting nurse demand in South Korea relies on the “health demands” method. This approach can also be viewed as a workload approach [12], as it initially estimates total health care utilization from patient census data, converting it to nurse workload by considering the ratio of nurses across health care institutions. Ultimately, nurse demand is estimated by dividing it by nurse productivity (or the nurse-to-patient ratio). Although this forecasting methodology can provide precise and detailed predictions by comprehensively considering the labor market’s structure, it has limitations such as the need for extensive data, complex model settings, and increased prediction errors in the face of technological or labor market changes.

Recent research on nurse supply and demand forecasts has revealed significant discrepancies, resulting in conflicting results reported by researchers. These inconsistencies pose challenges for policy-makers and hospital managers [13], with the primary cause believed to lie in the demand forecasting process rather than in supply forecasting. Demand forecasting encounters greater methodological challenges due to the intricate variables affecting RN demand and the high uncertainty surrounding future changes. Difficulties arise, particularly in accurately measuring actual nurse demand. Theoretical limitations in nurse workforce demand forecasting include identification problems [12], such as challenges in distinguishing demand and supply curves. Demand and supply functions are influenced by endogenous factors, such as nurses’ wages and productivity, as well as exogenous factors, such as nursing aides’ supply and leisure time, necessitating complex simultaneous equation modeling. Additionally, employment levels may not accurately reflect workforce demand, as demand is only reflected when supply exceeds it. In other words, identifying demand and supply curves in reality is challenging, and resolving endogeneity between them is difficult. To obtain data for conceptual workforce demand measurements, forecasting studies are underway to recognize and analyze employment levels as labor demand in response to these theoretical and practical challenges [14, 15].

Failure to forecast RN demand has several side effects. For example, an underestimation of nurse demand indicates a relatively high actual nursing capacity, which can cause problems, such as excessive competition among nurses, low wages, and rising unemployment. Conversely, overestimated RN demand can have undesirable consequences, such as overworking nurses, degrading the quality of nursing services, and patient dissatisfaction. Therefore, to prevent the failure of nurse demand forecasting and to rationalize nurses’ human resources, the need to determine the relationship between the methodological characteristics of demand forecasting and forecast errors should be understood, and accurate forecasting research should be conducted.

Studies have reported findings on nurse demand and supply forecasts, but they have been insufficient for evaluating the forecasting of nurses. Post hoc tests of forecasting accuracy effectively help individuals understand forecasts and results, measure the availability of data, and make policy decisions [16, 17]. We aimed to assess forecasting accuracy and investigate its determinants through post-evaluation of demand forecasts reported in past studies in terms of demand–supply forecasting.

## Methods

### Design

This study used secondary data analysis in which we quantitatively examine existing forecasting methods for nursing staff demand, assess the forecasting accuracy for nursing staff demand, and explore the factors influencing forecast accuracy.

### Samples

**Fig. 1:**
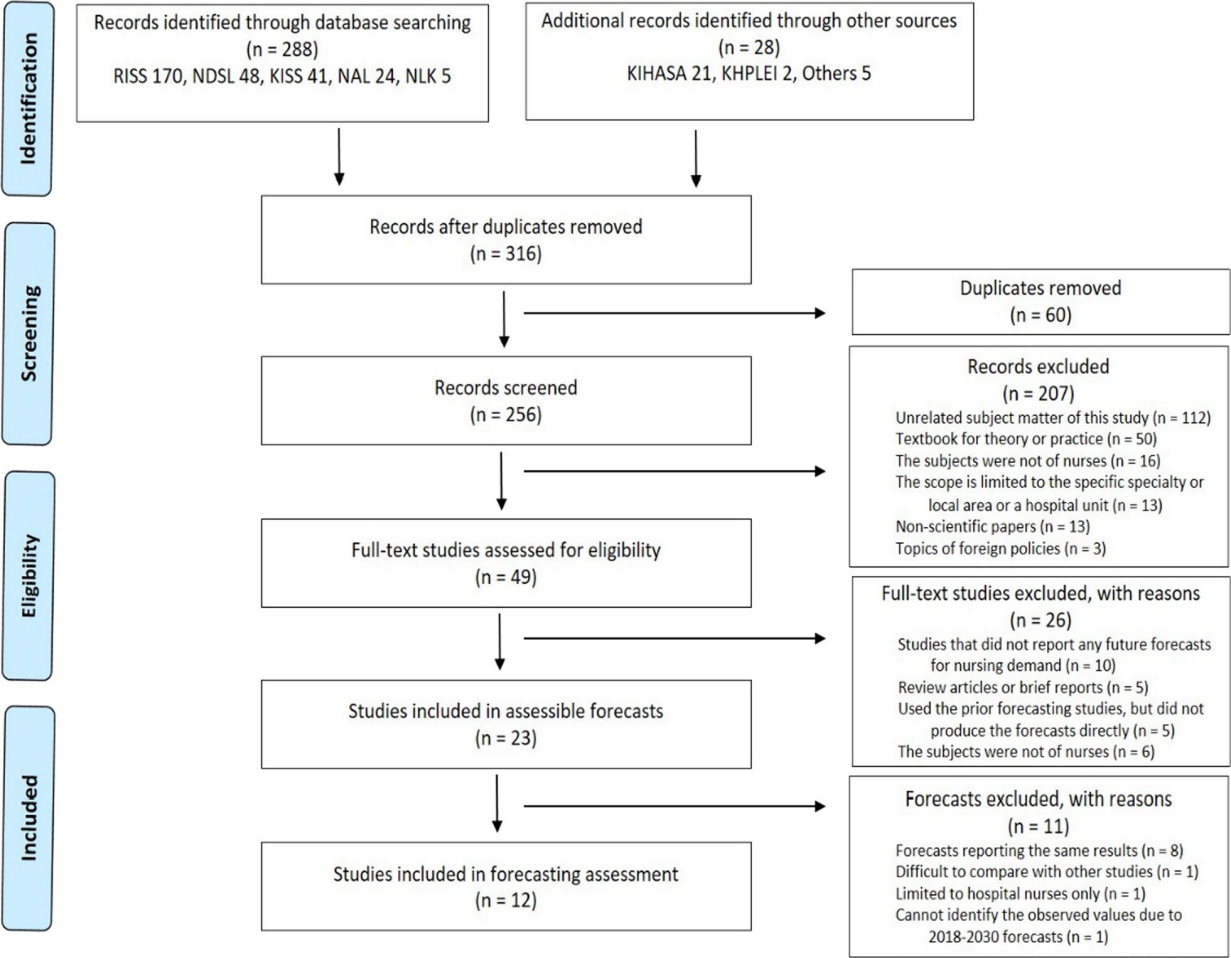
PRISMA flowchart. RISS,  Research Information Sharing Service; NDSL,  National Digital Science Library; KISS, Korean Studies Information Service System; NAL,  National Assembly Library; NLK, National Library of Korea; KIHASA,  Korea Institute for Health and Social Affairs; KHPLEI, Korea Health Personnel Licensing Examination Institute

The subject of this study is the predicted demand for nursing staff collected from previous workforce studies. The unit of analysis in this study is the forecast horizon and the predicted demand for nursing staff. The predicted demand for nursing staff refers to the estimated quantity of nursing personnel at a specific future point, as anticipated by forecasters in nursing workforce studies, representing the total nursing workforce required in the clinical sector at the national level. To collect predicted values, a systematic literature review was conducted targeting Korean domestic research reports and the academic literature on nursing workforce predictions. Subsequently, methodological information on workforce studies and predicted values for nursing staff demand were extracted from the selected literature. A systematic literature review was conducted per the Preferred Reporting Items for Systematic Reviews and Meta-Analyses (PRISMA) flowchart (Fig. 1), building on our published research [18]. A search was conducted from August 1 to August 31, 2017, using predetermined search terms (see Additional file 1). Of the 316 articles initially collected, 60 duplicates were removed, and 207 articles were excluded in the first round based on the literature selection criteria. The full texts of the 49 initially selected articles were obtained and reviewed, leading to the exclusion of 26 articles based on the literature selection criteria. The reasons for exclusion were as follows: 10 studies did not report nursing staff demand predictions, 5 were reviews or summary reports, 5 directly reported results cited from other studies, and 6 did not target nursing staff. A total of 23 literature sources were selected through this process. A detailed examination based on the testability of the predicted values revealed cases in which predictions were reported similarly across the literature and instances of studies with limited or unfeasible quantitative validation and comparability. After these 11 studies were excluded, a total of 12 studies were ultimately selected (see Additional file 2).

### Measures

#### Dependent variables

##### Actual and predicted demand for nursing staff

In this study, we investigated the actual and predicted demand for nursing staff, specifically focusing on clinical sectors. Nursing staff include those working in hospitals, clinics, and maternity hospitals, excluding public health institutions and nonclinical sectors. The nonclinical sector is excluded due to inconsistencies in definitions and challenges in data availability. Public health institutions are also excluded to maintain consistency with prior forecasting studies. The specific criteria are outlined as follows:

- The inclusion criteria were as follows: hospital-level health care institutions (general hospitals, hospitals, dental hospitals, Eastern medicine hospitals, etc.), clinic-level health care institutions (clinics, dental clinics, Eastern medicine clinics), and maternity hospitals.
- The exclusion criteria were as follows: public health institutions (public health centers, health subcenters and health clinics), nonclinical sectors of schools and research institutions, etc.

Actual demand is measured by the number of nurses employed in clinical sectors, whereas projected demand (forecasts) is derived from estimates from previous RN workforce studies. Selection criteria for forecasts entail a clearly defined forecasting period predating 2017, identifiable methodological details, and a focus on the clinical sector. Exclusion criteria include forecasts extending beyond 2017, lack of methodological clarity, ambiguous interpretation of findings, and forecasts related to the nonclinical sectors.

##### Forecasting accuracy

The forecasting accuracy was measured using the mean absolute percentage error (MAPE). The MAPE is a percentage obtained by dividing the absolute difference between the actual and predicted values by the actual value. Unlike the mean percentage error (MPE), the absolute value allows for an accurate assessment of the relative error magnitude without offsetting due to the sign of the predicted values. The MAPE disregards the direction of errors and serves as a measure of the prediction accuracy. A higher MAPE indicates lower forecasting accuracy, and a lower MAPE suggests more accurate forecasts. The specific calculation formula for the MAPE is as follows (1):

1 $${MAPE}_{ h}= \frac{100}{n}\sum_{t}\left|\frac{{A}_{t}-{F}_{t}}{{A}_{t}}\right|.$$

*h*, forecast horizon; *n*, number of forecasts within a forecast horizon; *t*, target year; *A*_*t*_, actual value at *t; F*_*t*_, predicted value at *t*.

##### Forecasting bias

The forecasting bias refers to the average direction of the errors in the predictions. In this study, forecasting bias was measured by the mean percentage error (MPE), which allows us to analyze the percentage errors at each forecasting point in the forecast horizon and determine the overall direction of the average error. A positive value (+) was interpreted as indicating pessimism bias, indicating that the actual values were higher than the predicted values in the case of nursing staff demand. Conversely, a negative value (−) was interpreted as optimistic bias, suggesting an underestimation of nursing staff demand for positive values and an overestimation of negative values. The specific calculation formula for MPE is as follows (2):

2 $${MPE}_{ h}= \frac{100}{n}\sum_{t}\frac{{A}_{t}-{F}_{t}}{{A}_{t}}.$$

*h*, forecast horizon; *n*, number of forecasts within a forecast horizon; *t*, target year; *A*_*t*_, actual value at *t; F*_*t*_, predicted value at *t*.

### Independent variables

The factors and variable measurements influencing forecasting accuracy are structured as shown in Table 1.

**Table 1:** Factor affecting forecast accuracy in this study

Factor	Variable	Measurement
Researcher factor	RN licensure of PI^1)^	• Non-RN (without RN licensure) = 0 • RN (with RN licensure) = 1
	Type of research institute	• University = 0 • Public institute = 1
	Type of funding agency	• Professional association = 0 • Public institute = 1
Methodological factor		
Set of assumption	Type of trend-fitting method	• Square root, logarithm = 0 • Growth rate, MA, logistic = 1 • ARIMA = 2
	RN productivity	• Current level = 0 • Medical law = 1 • Patient classification system = 2
	Annual workdays	• 255 days = 0 • 265 days = 1
Time frame	Length of data period	• 1–5 years = 0 • 6 –10 years = 1 • 11–15 years = 2
	Length of forecast horizon	• 1–5 years = 0 • 6–10 years = 1 • 11–15 years = 2 • 16–20 years = 3
Control variable	Launch year	• The period 1991–2000 • The period 2001–2015

PI, principal investigator; MA, moving average smoothing; ARIMA, auto regressive integrated moving average

#### Methodological factors

Nurse productivity is defined as the number of inpatients served by one nurse per day. In domestic RN workforce studies, common criteria for nurse productivity include standards set by medical laws (Law), current productivity levels (Current), and patient classification systems (PCS). In their forecasting studies, ‘nurse productivity’ is used interchangeably with concepts such as nursing workload, nursing intensity, and nurse-to-patient ratios, as seen in recent workforce studies. As shown in Table 2, the nursing workload based on ‘Current’ was greater than that based on ‘Law’, whereas the nursing workload based on ‘PCS’ was less than that based on ‘Law’. When comparing the nurse productivity criteria in this study to the methodological approaches regarding nurse workload and staffing proposed by Griffiths and colleagues [19], we note the following:

- Medical law criteria adopt a ‘benchmarking approach’, where one nurse cares for 2.5 inpatients daily, a normative standard in South Korea. These criteria equate the nursing workload of one inpatient to that of 12 outpatients.
- Current productivity criteria represent a ‘volume-based approach’, determining the required number of RNs based on patient volumes at the time of forecasting, utilizing patient census data. For instance, 'inpatient 5.0' indicates that one nurse cares for 5.0 inpatients daily. Converted to an 8-h shift, considering the RN’s shift and off schedules, approximately one nurse cares for 20 inpatients.
- Patient classification systems utilize the 'patient prototype approach', grouping patients based on nursing care needs and assigning a required staffing level for each group. This classification is based on unit or hospital-level patient dependency or acuity in the Korean context, categorizing patients into four groups with assigned weightings indicating the required staff associated with patients in each category, similar to The Safer Nursing Care Tool in the UK.

**Table 2:** Descriptive characteristics for forecasting assessment in included studies

No.	PI	Researcher factor			Methodological factor										
		RN licensure of PI	Type of research institute	Type of funding Agency	Launch year	Number of forecasts (n = 196)	Number of forecast horizons (n = 105)	Time frame		Trend-fitting method		RN productivity		Annual workdays	
								Length of data period	Length of forecast horizon	Number of methods	Details	Number of assumptions	Details	Number of assumptions	Details
1	Kim ES	RN	University	Public institute	1991	20	5	1984–1990	1991–2010	1	Logarithm	5	(Current) Hospital—inpatient 4.6; Clinic—inpatient 31.9 (Law) Hospital—inpatient 2.5; Clinic—inpatient 2.5 (PCS) Hospital—inpatient 1.5; Clinic—inpatient 1.5 (Law) Hospital—inpatient 2.5; Clinic—inpatient 31.9 (PCS) Hospital—inpatient 1.5; Clinic—inpatient 31.9	1	265
2	Park HY	RN	University	Professional association	1993	12	3	1984–1991	1993–2010	1	Logarithm	3	(Current) inpatient 5(= outpatient 60) (Law) inpatient 2.5(= outpatient 30) (PCS) inpatient 1.5(= outpatient 40)	1	265
3	Kim HJ	RN	University	Professional association	1996	15	5	1985–1994	1996–2010	1	SQRT	5	(Current) inpatient 5.5 (Law) inpatient 2.5 (PCS) inpatient 2.1 and intensive care patient 0.5 (Law) inpatient 2.5 (only hospital) (PCS) inpatient 2.1 and intensive care patient 0.5 (only hospital)	1	265
4	Choi EY	Non-RN	Public institute	Public institute	1998	12	4	1990–1997	1998–2012	1	SQRT	2	(Current) inpatient 4(= outpatient 45) (Law) inpatient 2.5(= outpatient 30)	2	255, 265
5	Park HY	RN	University	Professional association	1998	12	3	1989–1995	1998–2015	1	SQRT	3	(Current) inpatient 4(= outpatient 45) (Law) inpatient 2.5(= outpatient 30) (PCS) inpatient 1.5(= outpatient 40)	1	265
6	Park HY	RN	University	Professional association	2002	9	3	1990–1999	2002–2020	1	SQRT	3	(Current) inpatient 5(= outpatient 60) (Law) inpatient 2.5(= outpatient 30) (PCS) inpatient 1.5(= outpatient 40)	1	265
7	Lee SY	Non-RN	Public institute	Public institute	2003	8	4	1990–2002	2003–2018	1	Logistic	2	(Current) inpatient 4(= outpatient 45) (Law) inpatient 2.5(= outpatient 30)	2	255, 265
8	Jo JG	Non-RN	Public institute	Public institute	2005	8	4	1990–2003	2005–2018	1	Logistic	2	(Current) inpatient 4(= outpatient 45) (Law) inpatient 2.5(= outpatient 30)	2	255, 265
9	Oh YH	Non-RN	Public institute	Public institute	2006	8	4	2001–2004	2006–2020	1	Average growth rate	2	(Current) inpatient 4(= outpatient 45) (Law) inpatient 2.5(= outpatient 30)	2	255, 265
10	Yang DH	Non-RN	University	Professional association	2009	4	2	2004–2007	2009–2020	1	MA	1	(Current) inpatient 5.4(= outpatient 45.7)	2	255, 265
11	Oh YH	Non-RN	Public institute	Public institute	2010	40	20	2003–2007	2010–2025	2	1) Logistic 2) ARIMA	5	(Current) outpatient 63.3 (Current) outpatient 58.1 (Current) outpatient 52.8 (Current) outpatient 47.5 (Current) outpatient 42.2	2	255, 265
12	Oh YH	Non-RN	Public institute	Public institute	2014	48	48	2003–2012	2015–2030	4	1) Average growth rate 2) Logistic 3) Logarithm 4) ARIMA	6	(Current) outpatients 67.0 (Current) outpatients 61.4 (Current) outpatients 55.8 (Current) outpatients 50.2 (Current) outpatients 44.6 (Current) inpatient 2.5(= outpatient 30)	2	255, 265

PI, principal investigator; SQRT, square root method; MA, moving average method; ARIMA, autoregressive integrated moving average

When referring to the details of RN productivity, ‘Current’ indicates the current productivity levels, ‘Law’ represents the standards set by medical laws, and ‘PCS’ indicates the standards set by the patient classification system

Nurses’ annual workdays represent the number of working days in a year during which nurses provide patient care. In this study, 255 and 265 annual workdays, which are frequently used by nursing workforce researchers, were used.

The trend-fitting method defines a trend function that expresses the pattern of past time series data as a function of time when estimating medical utilization. The types of trend-fitting methods used in this study were broadly categorized into three groups based on trend models proposed in individual workforce studies: (1) medical utilization increasing gradually or becoming saturated at a certain point (square root, log model); (2) medical utilization increasing linearly or with a rapidly increasing segment (average growth rating, moving average method, logistic model); and (3) medical utilization changing probabilistically (ARIMA = autoregressive integrated moving average).

The length of the data period refers to the temporal duration of the historical data used for model identification and estimation. In this study, the length of the data period was calculated by measuring the temporal range of the time series data used for medical utilization estimation in previous nursing workforce studies (the final year minus the starting year of past data).

The length of the forecast horizon is defined as the interval between the point of prediction generation and the target point of prediction. In this study, the length of the forecast horizon was defined as the difference between the target year and the launch year. The launch year (year of prediction generation) was categorized into the period 1991–2000 and the period 2001–2015. The launch year was considered a controlled variable in this study to control for the time effect caused by the prediction generation year through subgroup analysis. Although there is no theoretical basis for setting 2000 as the cutoff year, it was chosen because there is a risk of lower prediction accuracy for forecasts produced in the past. Additionally, considering the overall realization rate of predictions and the point at which prominent forecasters changed, studies were broadly categorized into ‘studies before 2000’ and ‘studies after 2000’. The publication year was included to maintain consistency in the analysis because some studies had unclear baseline years. The target year is the estimated point at which the prediction is expected to materialize. Finally, the lengths of the forecast horizons were categorized as 1–5 years, 6–10 years, 11–15 years, and 16–20 years.

#### Researcher factors

In this study, researcher-related variables included whether the principal investigator held a nursing license, the type of research institution, and the type of funding agency. The possession of a nursing license by the principal investigator, who led the workforce study, was used as the criterion to define whether the investigator held a nursing license. The research institutions were categorized as *universities* or *public institutions* based on the nature of the research institution specified in the reports or academic journals. The funding agencies were classified as *professional associations*, such as the Korean Nurses Association and the Korean Medical Association, and as *public institutions*, such as the Korea Institute for Health and Social Affairs and the Korea Health Personnel Licensing Examination Institute.

### Statistical analysis

To analyze the differences in forecasting accuracy among factors, the Mann‒Whitney U test was used to determine the MAPE with two groups at the factor level, whereas the Kruskal‒Wallis H test was used to determine the MAPE with three or more groups. In cases in which the Kruskal‒Wallis H test statistic was significant, the significance level for pairwise comparisons in post hoc tests was determined using Bonferroni correction to establish the rejection region. The correlation between subvariables within the researcher and methodological factors was tested using Cramer's V. Cramer's V ranges from 0 to 1, where a value closer to 1 indicates a stronger correlation between variables. Forecasting bias was analyzed using the Chi-square test to determine relevance. The level of statistical significance was set at 5%.

## Results

### Methodological characteristics of the included studies

Table 2 presents the methodology and key assumptions for predicting nursing staff demand, as identified in the selected literature. We generally determined the methodology for forecasting nursing staff demand by estimating health care utilization and assumptions about nursing staff productivity. All 12 studies utilized a health care utilization-based approach, estimating health care demand through the analysis of trends in hospitalization and outpatient days. Although there were minor differences over time, researchers tended to use similar analysis techniques. Trend-fitting methods include curve estimation methods, such as logarithmic and logistic functions with the square root model, obtained by transforming the year into its square root; these methods are widely applied. Additionally, there were variations among researchers, ranging from the simplest form of the average-growth-rate method to more complex statistical knowledge required for time series analysis using the ARIMA model. Assumptions regarding annual workdays shifted from a historical single assumption of 265 days to an adoption of multiple assumptions of 255 and 265 days. The assumptions regarding nursing staff productivity involved multiple assumptions in all studies but one, utilizing three productivity assumptions per study.

### Forecasting accuracy of the included studies

The forecast horizon, the unit of analysis in this study, comprised 105 periods, with an average MAPE of 34.8% (Table 3). The median was 20.3%, and the values ranged from 0.2% to 191.3%. Twenty forecast horizons were collected from five studies conducted before 2000, whereas 85 forecast horizons were collected from seven studies conducted after 2000, indicating a roughly fourfold difference in sample size. This discrepancy can be attributed to the research trend in the 2000s, in which multiple predictions were reported by combining various analytical assumptions and techniques. Overall, studies conducted before 2000 exhibited lower forecasting accuracy than those conducted after 2000. According to the Mann‒Whitney U test results, there was a significant difference in the MAPE based on the launch year.

**Table 3:** Forecasting accuracy of included studies

Classification	FH	MAPE (%)						
	Total	Mean	SD	U(*p*)^a^	min	max	Median	IQR
Total	105	34.8	38.0		0.2	191.3	20.3	33.5
Launch year								
1991–2000 studies	20	61.3	51.4	449.00 (0.001)	6.8	191.3	47.6	70.3
2001–2015 studies	85	28.6	31.5		0.2	136.5	17.1	24.3

FH, forecast horizon; MAPE,  mean absolute percentage error; SD, standard deviation; IQR,  interquartile range

^a^Mann–Whitney U test

### Forecasting bias of the included studies

**Fig. 2:**
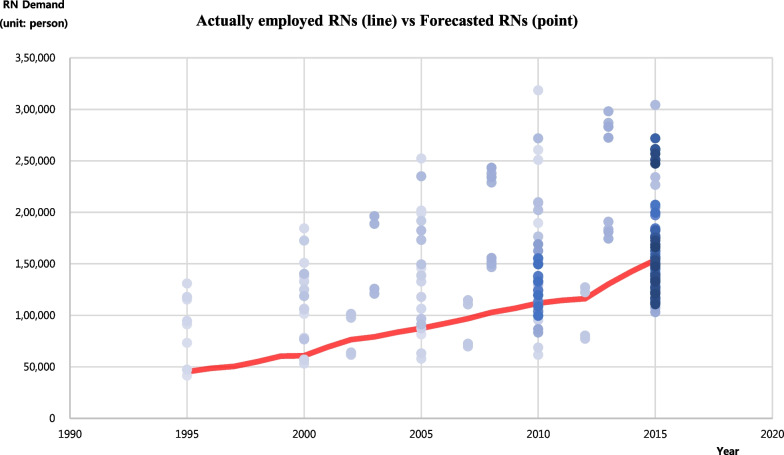
Comparison between actual and forecasted RNs demand

A significant portion of the nursing staff demand predictions (points) were distributed above the actual demand (line), as shown in Fig. 2. Overall, 41.9% of the predictions were positive ( +), and 58.1% were negative (−), indicating a tendency toward overestimation. However, when controlling for the launch year, we detected no significant difference between the positive ( +) and negative (−) predictions (χ^2^ = 2.90, p = 0.089).

### Association between researcher factor and methodological factor

The analysis of the correlation between subvariables of the researcher and methodological factors revealed significant correlations among all variables except for the relationship between the type of funding agency and the length of the data period (Table 4). Particularly in terms of trend-fitting methods, principal investigators (PIs) with RN licenses only used square root and logarithmic models, estimating future health care utilization reaching a saturation point, whereas those without RN licenses employed a variety of other techniques. Furthermore, concerning assumptions about nurse productivity, PIs with RN licenses primarily utilized patient classification systems as productivity assumptions, whereas non-RN researchers did not use these criteria at all; instead, they relied mainly on current productivity assumptions.

**Table 4:** Association between researcher factor and methodological factor

Methodological factor	FH total	Researcher factor														
		RN licensure of PI					Type of research institute					Type of funding agency				
		Non-RN		RN		Cramer V (*p*)	University		Public institute		Cramer V (*p*)	Professional association		Public institute		Cramer V (*p*)
		n	%	n	%		n	%	n	%		n	%	n	%	
RN productivity																
Current level	75	70	93	5	7	0.62 (< 0.001)	7	9	68	91	0.58 (< 0.001)	6	8	69	92	0.45 (< 0.001)
Law	23	16	70	7	30		7	30	16	70		5	22	18	78	
Patient classification system	7	0	0	7	100		7	100	0	0		5	71	2	29	
Annual workdays																
255 days	43	43	100	0	0	0.39 (< 0.001)	1	2	42	98	0.37 (< 0.001)	1	2	42	98	0.30 (0.002)
265 days	62	43	69	19	31		20	32	42	68		15	24	47	76	
Trend-fitting method																
Square root, logarithm	35	16	46	19	54	0.67 (< 0.001)	19	54	16	46	0.61 (< 0.001)	14	40	21	60	0.49 (< 0.001)
Growth rate, MA, logistic	48	48	100	0	0		2	4	46	96		2	4	46	96	
ARIMA	22	22	100	0	0		0	0	22	100		0	0	22	100	
Length of past data																
1–5 years	26	26	100	0	0	0.33 (0.004)	2	8	24	92	0.25 (0.039)	2	8	24	92	0.19 (0.158)
6–10 years	71	52	73	19	27		19	27	52	73		14	20	57	80	
11– 15 years	8	8	100	0	0		0	0	8	100		0	0	8	100	
Length of forecast horizon																
1–5 years	68	68	100	0	0	0.91 (< 0.001)	0	0	68	100	0.86 (< 0.001)	0	0	68	100	0.70 (< 0.001)
6–10 years	14	14	100	0	0		2	14	12	86		2	14	12	86	
11–15 years	12	4	33	8	67		8	67	4	33		8	67	4	33	
16–20 years	11	0	0	11	100		11	100	0	0		6	55	5	45	

FH, forecast horizon; PI, principal investigator; MA,  moving average smoothing; ARIMA, auto regressive integrated moving average

### Analysis of forecasting accuracy by factor

When we examined forecasting accuracy without controlling for the launch year, there were significant differences in forecasting accuracy among trend-fitting methods for the methodological factors and among all variables for the researcher factors (Table 5). However, when we controlled for the launch year, these differences were no longer significant. Variables such as nursing staff productivity, length of the data period, and length of forecast horizon maintained their significance even after we controlled for the launch year. These variables are considered crucial factors influencing the accuracy of nursing staff demand predictions.

**Table 5:** Forecast accuracy by methodological and researcher factor

Factor	Total studies				Launch year							
					1991–2000 studies				2001–2015 studies			
	FH	MAPE (%)		*H or U* (*p*)	FH	MAPE (%)		*H or U* (*p*)	FH	MAPE (%)		*H or U* (*p*)
	Total	Mean	SD		Total	Mean	SD		Total	Mean	SD	
**Methodological factor**												
RN Productivity												
Current level^a^	75	16.9	11.4	45.88	6	20.0	7.9	9.50	69	16.6	11.6	29.14
Law^b^	23	69.0	38.2	(< 0.001)	8	55.0	41.2	(0.009)	15	76.5	35.8	(< 0.001)
Patient classification system^c^	7	114.6	46.9	b,c > a	6	111.0	50.2	c > a	1	136.5	-	b > a
Annual workdays												
255 days	43	27.2	29.7	1,550.50	2	22.2	2.7	27.00	41	27.5	30.5	930.50
265 days	62	40.1	42.3	(0.156)	18	65.6	52.4	(0.316)	44	29.6	32.7	(0.802)
Type of trend-fitting method												
Square root, logarithm^a^	35	49.3	47.6	7.84	12^1), *^	44.1^1), *^	36.3^1), *^	67.00	15	33.4	38.1	
Growth rate, MA, logistic^b^	48	31.5	33.7	(0.020)	8^1), †^	87.0^2), †^	62.0^2), †^	(0.157)	48	31.5	33.7	2.14
ARIMA^c^	22	19.0	17.6	a > c	–	–	–	–	22	19.0	17.6	(0.344)
Length of data period												
1–5 years^a^	26	17.1	11.5	15.51	8^2), ‡^	86.5^2), ‡^	62.9^2), ‡^		26	17.1	11.5	16.23
6–10 years^b^	71	35.8	39.0	(< 0.001)	7^2), §^	36.7^2), §^	27.4^2), §^	1.79	51	25.8	27.7	(< 0.001)
11–15 years^c^	8	83.8	43.3	c > a,b	5^2), ||^	55.3^2), ||^	46.1^2), ||^	(.410)	8	83.8	43.3	c > a,b
Length of forecast horizon												
1–5 years^a^	68	20.3	19.5		–	–	–	–	68	20.3	19.5	
6–10 years^b^	14	58.5	44.3	28.90	–	–	–	–	14	58.5	44.3	17.53
11– 15 years^c^	12	49.5	44.7	(< 0.001)	9	40.4	37.2	66.00	3	76.9	62.8	(< 0.001)
16–20 years^d^	11	78.4	56.5	b,c,d > a	11	78.4	56.5	(.230)	–	–	–	b > a
**Researcher factor**												
RN licensure of PI												
RN	86	26.6	28.3	1272.00	4	21.7	4.9	49.00	82	26.8	29.0	186.00
Non-RN	19	72.1	52.8	(< 0.001)	16	71.1	53.1	(0.122)	3	76.9	62.8	(0.144)
Type of research institute												
University	21	68.3	51.5	388.00	16	71.1	53.1	15.00	5	59.1	50.6	96.00
Public institute	84	26.4	28.6	(< 0.001)	4	21.7	4.9	(0.122)	80	26.7	29.3	(0.052)
Type of funding agency												
Professional association	16	56.8	40.1	364.00	11	59.1	50.6	50.00	5	59.1	50.6	96.00
Public institute	89	30.9	36.5	(0.002)	9	68.1	66.7	(1.000)	80	26.7	29.3	(0.052)

FH, forecast horizon; MAPE, mean absolute percentage error; SD, standard deviation; H, Kruskal–Wallis H test statistics; U,  Mann–Whitney U test statistics; MA, moving average smoothing; ARIMA, autoregressive integrated moving average

1) Type of trend-fitting method for 1991 ~ 2000 studies

2) Length of data period for 1991 ~ 2000 studies

^*^Square root; ^†^ logarithm

^‡^6 years

^§^7 years

^||^9 years

## Discussion

Over the past 25 years, the policy environment regarding the nursing workforce in South Korea has undergone rapid changes. Nursing workforce forecasting studies function as essential input variables, playing a crucial role in the rational decision-making process for workforce policies. This study contributes to the improvement of nursing workforce studies and holds importance for evaluating nursing workforce policies. In this study, we conducted a detailed review of the methodological aspects of nursing workforce studies. We analyzed 105 forecast horizons extracted from existing nursing workforce studies, and the results revealed an average MAPE of 34.8%, with an overestimation rate of 58.1%, which was slightly greater than the underestimation rate. Factors influencing forecasting accuracy included assumptions about nursing productivity, the length of the data period, and the length of the forecast horizon. Although there were significant differences in the researcher factors and trend-fitting methods before we controlled for the launch year, we observed no significant differences after the control. The assumptions about annual working days showed no significant association with forecasting accuracy.

According to our findings, considering the assumption of nursing staff productivity using the current productivity criteria at the time of prediction, the MAPE was 16.9%, which was significantly greater than the MAPEs for the Law criteria (69.0%) and the Patient Classification System criteria (114.6%). These results can be interpreted in two ways. First, the actual nursing staff productivity levels applied in nursing staff demand and supply studies over the past 25 years have consistently been higher than the criteria set by the Law or the Patient Classification System. Second, predictions based on the criteria of the Law or the Patient Classification System significantly exceeded the actual demand for nursing staff. It can be inferred that predictions based on the Law criteria may be unrealistic due to their low forecasting accuracy. Therefore, evaluating nursing staff demand using the current productivity criteria appears to be the most rational approach.

The rationale for the government’s adjustment of nursing school enrollment quotas is based on the prediction that the demand for nurses in the future will increase significantly. It can be assumed that the application of productivity based on the Law criteria played a role in such demand predictions. When the current productivity criteria are applied, a mixed pattern of shortages and surpluses arises, whereas applying the Law criteria results only in shortages. This can create a cycle in which the demand for nurses is determined by unrealistic demand predictions, leading to an expansion of supply. Therefore, setting productivity based on the Law criteria and the decision to expand supply accordingly have the potential to further distort the structure of the nursing labor market. In the nursing labor market, an increase in the supply of nurses is tied to lower wages, and health care institutions can continuously claim chronic shortages. Additionally, policies to address the shortage of nurses are disproportionately focused on adjusting enrollment quotas, and such adjustments are based on incorrect demand predictions. To generate a legally or policy-desirable level of demand for nurses, it is essential to address the current understaffing rate in health care institutions and apply rigorous staffing standards. For policy-makers seeking to resolve the issue of nursing shortages, according to the results of the system dynamics simulation model Murphy et al. [20] presented, an increase in nursing productivity was shown to be the most effective policy measure compared to increases in enrollment quotas, nurse retention, reduction in dropout rates, and reduction in absenteeism.

The approach of adopting assumptions about nursing productivity in existing studies is somewhat akin to a judgmental technique. The Law criteria or indicators used by previous demand and supply researchers have been conventionally applied, and these methods are more based on intuition than are scientific methods, making it difficult to achieve rationality and reliability. Depending on the researcher, this approach carries the risk of arbitrarily making strategic decisions. A study reporting health care workforce estimates for 18 countries highlighted the issue of arbitrary productivity [21]. Productivity improvement is a topic that is often overlooked in workforce planning or estimation models; through improvements in nursing productivity, demand can be adequately met with fewer nurses [22]. Nursing productivity is influenced by factors such as the safety of the work environment, the availability of support staff and services, efficient nurse deployment, and nurses’ abilities (skills, knowledge, and judgment). An increase in nursing productivity means working more productively and efficiently rather than working harder or longer [20]. Nursing workforce polices are needed to encourage the establishment of realistic criteria for nursing productivity and accurate measurements of nursing productivity.

This study’s results showed a significant difference in forecasting accuracy among the forecasting methods only when we did not control for the launch year. Nevertheless, it is worth discussing the ARIMA model’s superior forecasting accuracy (assuming that medical utilization changes probabilistically) compared to that of the logarithmic and square root models (assuming that medical utilization increases gradually or becomes saturated at a certain point). However, ARIMA predictions are preferable to short-term predictions because ARIMA’s prediction confidence interval gradually expands over time, increasing the efficiency of short-term predictions with high volatility but decreasing the favorability of long-term predictions [23, 24]. Fundamentally, for long-term predictions, it is advisable to develop forecasting methods that can proactively respond to future shocks, such as scenario modeling. In advanced countries, efforts are made to avoid traditional time series analysis methods and to make supply predictions by considering various scenarios [25].

In this study, the relationship between the length of the data period and the forecasting accuracy reveals that as the length of the data period increases, the forecasting accuracy decreases. This contradicts previous studies’ findings because the general understanding is that the relationship between the length of the data period and forecasting accuracy is weak but positive [26–28]. There are two possible causes for this discrepancy. First, due to the short-term fluctuations in health care utilization over the past few years, there is a risk of underestimating parameters with longer historical data periods. Second, because there was a high correlation between researcher-related factors and methodological factors in this study, the results should not be interpreted as exclusively the effect of the data period.

The forecasting accuracy is greater for forecasting horizons of 1 to 5 years than for longer periods. This finding aligns with the majority of previous studies, indicating that as the forecasting horizon increases, the forecasting accuracy tends to decrease [28, 29]. In supply studies, researchers have attempted to forecast health care workforce needs uniformly for more than 10 years. However, doubts remain as to whether such an approach effectively captures the differences in workforce structure and education duration across individual occupational sectors. Occupations requiring training periods of more than 10 years, such as physicians, dentists, and traditional Korean medicine doctors, may be suitable for medium- to long-term predictions. Nevertheless, for nursing professionals, who typically require 4 to 5 years of education and training, treating the forecasting period identically to that of physicians may not be necessary. According to evaluations of workforce predictions, accurate forecasting over 10 to 15 years is considered challenging, and such predictions are not particularly useful for educational decision-making [30]. Therefore, a reassessment of the forecasting period setting is necessary for nursing demand predictions. In addition, because longer forecasting periods result in lower forecasting accuracy, regular updates of supply information are needed.

The difference in forecasting accuracy according to researcher factors varied significantly depending on whether the launch year was controlled. This result is not unrelated to the high correlation among the subvariables of researcher factors and methodological factors. When the launch year was not controlled for, responsibility researchers holding nursing licenses, university-affiliated research institutions, and research funding agencies affiliated with professional associations had lower forecasting accuracy than their counterparts (those without nursing licenses, public institutions, and those affiliated with governmental agencies, respectively). However, when the launch year was controlled for, no significant differences were found in any of the researcher factors. Even though the researcher’s affiliation affects the forecasting accuracy [31], it is challenging to judge researchers’ bias exclusively based on whether they hold nursing licenses and whether researchers belonging to the same professional group reflect conflicts of interest. Ascher [32] theoretically explained the concept of “assumption drag”, which contributes to decreased forecasting accuracy. This concept refers to the tendency to persistently use existing assumptions, even when the assumptions introduced by forecasters lose their practical validity. Despite the loss of validity, analysts conventionally maintain their initial assumptions, leading to decreased forecasting accuracy. Experts in the forecasting field may be more inclined to be immersed in their own domain rather than maintain objective neutrality. Even when assumptions are clearly erroneous, they may implicitly or explicitly rely on their past assumptions. Therefore, forecasters should critically examine outdated assumptions and consider attempting new approaches in forecasting research.

This study has several limitations. First, the collection of predicted values from selected workforce literature resulted in data predominantly from only two studies, accounting for approximately 65% of the total prediction periods. The sample skewness issues weakened the results’ internal validity. Second, although we conducted a systematic literature review, there may be instances of missing data even if highly relevant to this study. Because workforce research has been predominantly conducted by public institutions or professional associations rather than academic institutions, there were restrictions regarding the availability of data. Third, constraints were noted in reproducing the demand predictions estimated in workforce studies due to inadequate descriptions of the original data and data sources used by analysts at that time or difficulty in understanding the ambiguous technical methods employed in the demand estimation process.

## Conclusions

The assumption regarding nurse productivity has been recognized as a major factor contributing to inaccuracies in nursing demand forecasts. Even minor adjustments of nurse productivity in initial settings can greatly influence forecasting precision. Therefore, careful consideration is necessary when conducting studies on supply and demand forecasts or interpreting their outcomes. To enhance the accuracy of nurse demand forecasts, future research should focus on precisely measuring nursing workload and productivity in alignment with real-world situations.

### Supplementary Information

Supplementary Material 1.

Supplementary Material 2.

## Data Availability

Data will be made available from the corresponding author on reasonable request.
