# Supplementary material for: Forecast accuracy of demand for registered nurses and its determinants in South Korea
Source: Hum Resour Health. 2024 Jun 25;22:44. doi: 10.1186/s12960-024-00910-3 (PMC11197229; doi:10.1186/s12960-024-00910-3)
Supplement: Supplementary file 1 — Supplementary Material 1. [file 12960_2024_910_MOESM1_ESM.docx]

**Predetermined search terms and results by database (English)**

| **Database** | **Search terms** | **Search results** |
| --- | --- | --- |
| RISS | - P: RN workforce OR nursing workforce OR health workforce - I: forecast OR projection OR foresight - O: demand OR supply and demand - Publication year : 1990-2017 | 170 |
| KISS | - P: RN workforce│nursing workforce│health workforce - I: forecast│projection│foresight - O: demand│supply and demand - Publication year : 1990-2017 | 41 |
| NDSL | Document type : articles, reports, proceedings   - P: RN workforce│nursing workforce│health workforce - I: forecast│projection│foresight - O: demand│supply and demand - Publication year : 1990-2017 | 48 |
| NAL | - P: RN workforce OR nursing workforce OR health workforce - I: forecast OR projection OR foresight - O: demand OR supply and demand - Publication year : 1990-2017 | 24 |
| NLK | - Examine in the search field such as title, author, publisher, keyword - Search by manual labor with combining different keywords (RN workforce, nursing workforce, health workforce, forecast, projection, foresight, demand, supply and demand) | 5 |
| KIHASA | - “RN workforce”, “nursing workforce”, “health workforce” | 21 |
| KHPLEI or others | - "Searching reports" Menu - "forecast" only | 7 |

Note. P=Population, I=intervention, O=outcomes, RN=registered nurse, RISS=Research Information Sharing Service, KISS=Korean Studies Information Service System, NDSL=National Digital Science Library, NAL=National Assembly Library, NLK=National Library of Korea, KIHASA= Korea Institute for Health and Social Affairs, KHPLEI=Korea Health Personnel Licensing Examination Institute.

**Predetermined search terms and results by database (Korean)**

| **Database** | **Search terms** | **Search results** |
| --- | --- | --- |
| RISS | - P: 간호사인력 <OR> 간호인력 <OR> 보건의료인력 - I: 추계 <OR> 예측 <OR> 전망 - O: 수요 <OR> 수급 - 발행연도: 1990-2017 | 170 |
| KISS | - P: 간호사인력│간호인력│보건의료인력 - I: 추계│예측│전망 - O: 수요│수급 - 발행연도: 1990-2017 | 41 |
| NDSL | 논문, 보고서, 저널프로시딩 중에서   - P: 간호사인력│간호인력│보건의료인력 - I: 추계│예측│전망 - O: 수요│수급 - 발행연도: 1990-2017 | 48 |
| NAL | - P: 간호사인력 OR 간호인력 OR 보건의료인력 - I: 추계 OR 예측 OR 전망 - O: 수요 OR 수급 - 발행연도: 1990-2017 | 24 |
| NLK | - 검색필드에 제목, 저자, 발행인, 키워드 등 검색 - 다음의 키워드들을 조합하여 수작업 검색(간호사인력, 간호인력, 보건의료인력, 추계, 예측, 전망, 수요, 수급) | 5 |
| KIHASA | - "간호사인력, 간호인력, 보건의료인력" only | 21 |
| KHPLEI  및 기타 | - "연구보고서 조회" menu - "추계" only | 7 |

Note. P=Population, I=intervention, O=outcomes, RN=registered nurse, RISS=Research Information Sharing Service, KISS=Korean Studies Information Service System, NDSL=National Digital Science Library, NAL=National Assembly Library, NLK=National Library of Korea, KIHASA= Korea Institute for Health and Social Affairs, KHPLEI=Korea Health Personnel Licensing Examination Institute.
