# Supplementary material for: Forecast accuracy of demand for registered nurses and its determinants in South Korea
Source: Hum Resour Health. 2024 Jun 25;22:44. doi: 10.1186/s12960-024-00910-3 (PMC11197229; doi:10.1186/s12960-024-00910-3)
Supplement: Supplementary file 2 — Supplementary Material 2. [file 12960_2024_910_MOESM2_ESM.docx]

**Finally included studies in the study through the systematic review (English title)**

1. Kim ES, Cho WJ, Cho WH, Lee CY, Ko IS, Jee SH, et al. Long term and short term public health manpower planning II: nurses, nurse aides, pharmacists, medical technicians. Seoul: Korea Institute for Health and Social Affairs; 1991.

2. Park HA, Choi YH, Ko IS, Lee SJ, Chang HS, Jeon CY. The supply and demand projection of nurses in Korea. Korean Nurs. 1993;32(3):52-67.

3. Kim HJ, Ko IS, Park HA, Lee SJ, Chang HS, Jeon CY. Mid- to long-term supply and demand plan for nursing workforce. Korean Nurses Association; 1996.

4. Park HA. Study on development of nursing workforce supply and demand plan (draft). Korean Nurses Association; 1996.

5. Park HA, Hyun SK, Han KJ, Park JH, Park SA. Analysis and projection of supply and demand for nursing workforce in Korea. Korean Nurse. 2002;41(1):51-68.

6. Choi EY, Jo JG, Kim JS, Lee WB. Prospects for supply and demand of medical personnel and policy issues: doctors, oriental doctors, dentists, nurses, and pharmacists. Korea Institute for Health and Social Affairs; 1996.

7. Lee SY, Oh YH, Song HJ, Kim EJ, Cho SH, Park JY, Jeong WJ. The present condition of supply and demand for healthcare resources and management policy implications; Korea Institute for Health and Social Affairs; 2003.

8. Jo JG, Lee SY, Kim EJ, Song HJ, Yoon KJ. A study on the demand for and supply of nurse manpower. Korea Institute for Health and Social Affairs; 2005.

9. Oh YH, Jo JG, Choi BH, Lee SH, Lee SY, Park JY, Park SK. Mid- to long-term estimates of medical supply. Korea Institute for Health and Social Affairs; 2006.

10. Yang DH, Jeong DC, Seo WS, Park HG. A study on the environmental change in Korean healthcare industry: redefining the role of healthcare professionals (MDs & Nurses) and hospitals. Research Institute for Healthcare Policy; 2009.

11. Oh YH, Jo JG, Kim JH, Ji YG. Mid- to long-term supply and demand forecasting of health care personnel. Korea Health Personnel Licensing Examination Institute; 2010.

12. Oh YH, et al. 2015-2030 Mid- to long-term supply and demand forecasting for health care workers. Korea Health Personnel Licensing Examination Institute; 2014.

**Finally included studies in the study through the systematic review (Korean title)**

1. 김의숙, 조원정, 조우현, 이정렬, 고일선, 지선하, 손태용(1991). 장단기 보건의료인력 수급에 관한 연구2: 간호사, 간호조무사, 약사, 의료기사(임상병리사, 방사선사, 물리치료사). 한국보건사회연구원.

2. 박현애, 최영희, 고일선, 이선자, 장현숙, 전춘영(1993). 우리나라 간호인력의 장기수급 대책: 2010년까지 수요 및 공급추계. 대한간호, 32(3), 52-67.

3. 김화중, 고일선, 박현애, 이선자, 장현숙, 전춘영(1996). 간호사 인력 중장기 수급계획. 대한간호협회.

4. 박현애 (1996). 간호인력 수급계획(안) 작성에 관한 연구. 대한간호협회.

5. 박현애, 현수경, 한경자, 박정호, 박성애(2002). 국내 간호인력 수급 분석 및 추계연구. 대한간호, 41(1), 51-68.

6. 최은영, 조재국, 김진수, 이우백(1996). 의약인력의 수급전망과 정책과제: 의사, 한의사, 치과의사, 간호사, 약사. 한국보건사회연구원.

7. 이상영, 오영호, 송현종, 김은정, 조성현, 박재용, 정우진(2003). 보건의료자원 수급 현황 및 관리정책 개선방안. 한구보건사회연구원.

8. 조재국, 이상영, 김은정, 송현종, 윤강재(2005). 간호사 인력의 수급추계와 정책과제. 한국보건사회연구원.

9. 오영호, 조재국, 최병호, 이신호, 이상영, 박재용, 박수경(2006). 의료공급중장기 추계. 한국보건사회연구원.

10. 양동현, 정두채, 서원식, 박효길(2009). 의료환경변화에 대한 연구: 의료인력 의사, 간호사 및 의료기관 기능 재정립. 의료정책연구소.

11. 오영호, 조재국, 김진현, 지영건(2010). 보건의료인력 중장기 수급추계연구. 한국보건의료인국가시험원.

12. 오영호, 유근춘, 도세록, 김진현, 지영건, 이난희, 김혜남(2014). 2015-2030 보건의료인 중장기 수급추계연구. 한국보건의료인국가시험원.
